# Distal femoral flexion deformity from growth disturbance treated with a two-level osteotomy and internal lengthening nail

**DOI:** 10.1007/s11751-017-0298-2

**Published:** 2017-10-16

**Authors:** Austin T. Fragomen, Fiona R. Fragomen

**Affiliations:** 1 Weill Medical College, Cornell University, 535 East 70th Street, New York, NY 10021 USA; 2 Hospital for Special Surgery, 535 East 70th Street, New York, NY 10021 USA

**Keywords:** Magnetic internal lengthening nail, Precice, TomoFix, Osteotomy, Deformity, Limb lengthening

## Abstract

Salter Harris fractures of the distal femur can lead to growth disturbance with resulting leg length inequality and knee deformity. We have looked at a case series (3) of patients who presented with a distal femur flexion malunion and shortening treated with a distal femoral osteotomy and plating and a proximal femoral osteotomy with a magnetic internal lengthening nail. Does a two-level osteotomy and internal fixation approach provide a reliable result both radiographically and functionally? The average knee extension loss was 12°, LLD 47 mm, PDFA 65°, MAD 2 mm. The patients were treated with an acute, posterior, opening wedge osteotomy of the distal femur stabilized with a lateral plate and screws and grafted with cancellous chips and putty. A second osteotomy was made proximally in the femur percutaneously, and the internal lengthening nail was inserted. Lengthening was done at approximately 1 mm/day. The average extension gain was 12°; amount of lengthening at the proximal site was 40 mm, LLD was 3 mm. The average PDFA was 81°, and MAD 3 mm. There were no complications. Functional results were excellent. Bone healing index was 24 days/cm. The average distance from the distal osteotomy to the joint line was 57 mm. The technique of two-level femur osteotomy stabilized with a plate and lengthening nail yielded excellent results with acceptable correction of deformity, full knee extension, and improved function. There were no complications including implant failure, infection, need for blood transfusion, knee stiffness, nonunion, compartment syndrome, or malunion.

## Introduction

Salter Harris fractures of the distal femur can lead to a high rate of growth disturbance [1]. The combination of the fracture and the physeal injury can result in leg length inequality and distal femoral deformity. Most deformity that is noticeable lies in the coronal plane with sagittal (flexion) deformity going underappreciated [1]. If diagnosed early, anterior physeal stapling can reduce the degree of flexion deformity [2, 3] but cannot correct the limb shortening. The apex of the flexion deformity, typically very close to the knee joint, influences treatment options. Anterior closing wedge distal femoral osteotomy with plate fixation has been used for treatment of knee flexion deformities in cerebral palsy [4] and polio [5] with success but at the expense of limb length [6]. Treatment by distal femoral osteotomy and a hexapod external fixator (or monolateral frame) can correct both deformity and length [7–10], but associated problems include knee stiffness [9, 11–14], pin infections [7–9], pain, difficulty sitting and sleeping, and difficulty controlling the sagittal plane with residual flexion deformity [8, 10, 15, 16]. External fixation has been shown to have a higher complication rate when compared with the internal lengthening nail (ILN) techniques [17]. Improved patient satisfaction with internal fixation has also pushed surgeons to seek surgical solutions without the use of external fixation [18]. The lengthening and then plating technique reduces time in the frame but is associated with plate failure, malunion, need for further surgery, and a risk of deep infection [19].

The ILN has solved many problems that were associated previously with external fixation. The problem with using the ILN and a single osteotomy approach to correct this distal femoral flexion deformity is that a very distal osteotomy (less than 7 cm from the joint line) is needed at apex of the deformity, making intramedullary (IM) nail fixation, lengthening over nail (LON), and ILN corrections less reliable. Furthermore, retrograde ILN is associated with risk of increased flexion deformity at the osteotomy site which would threaten to undo any correction initially obtained [20, 21]. Performing the osteotomy more proximally to allow for proper IM nail fixation would require excessive posterior translation of the distal fragment precluding IM nailing or, without the translation, result in under-correction of the flexion deformity.

We reviewed a case series of skeletally mature patients who presented with a distal femur flexion malunion and shortening. All patients were treated with a two-level osteotomy technique including a distal femoral osteotomy (DFO) using acute correction and plating and a proximal femoral osteotomy used for lengthening with a magnetic ILN. The question asked was: does a two-level osteotomy and all-internal fixation approach provide a reliable result both radiographically and functionally?

## Materials and methods

**Fig. 1:**
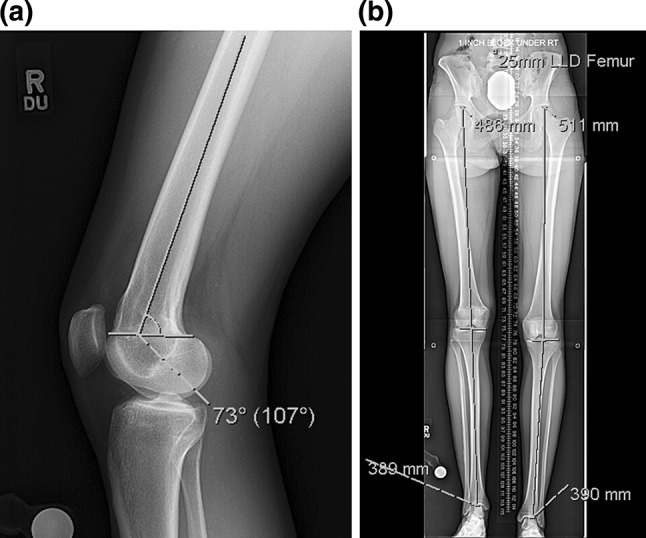
**a** This lateral radiograph of the knee shows a distal femoral flexion deformity of 10°. The posterior distal femoral angle (PDFA) measures 73° with the normal averaging 83°, **b** a 51 in., standing, bipedal radiograph with a 25-mm block under the right foot demonstrates a 25 mm leg length discrepancy originating from the right femur

**Fig. 2:**
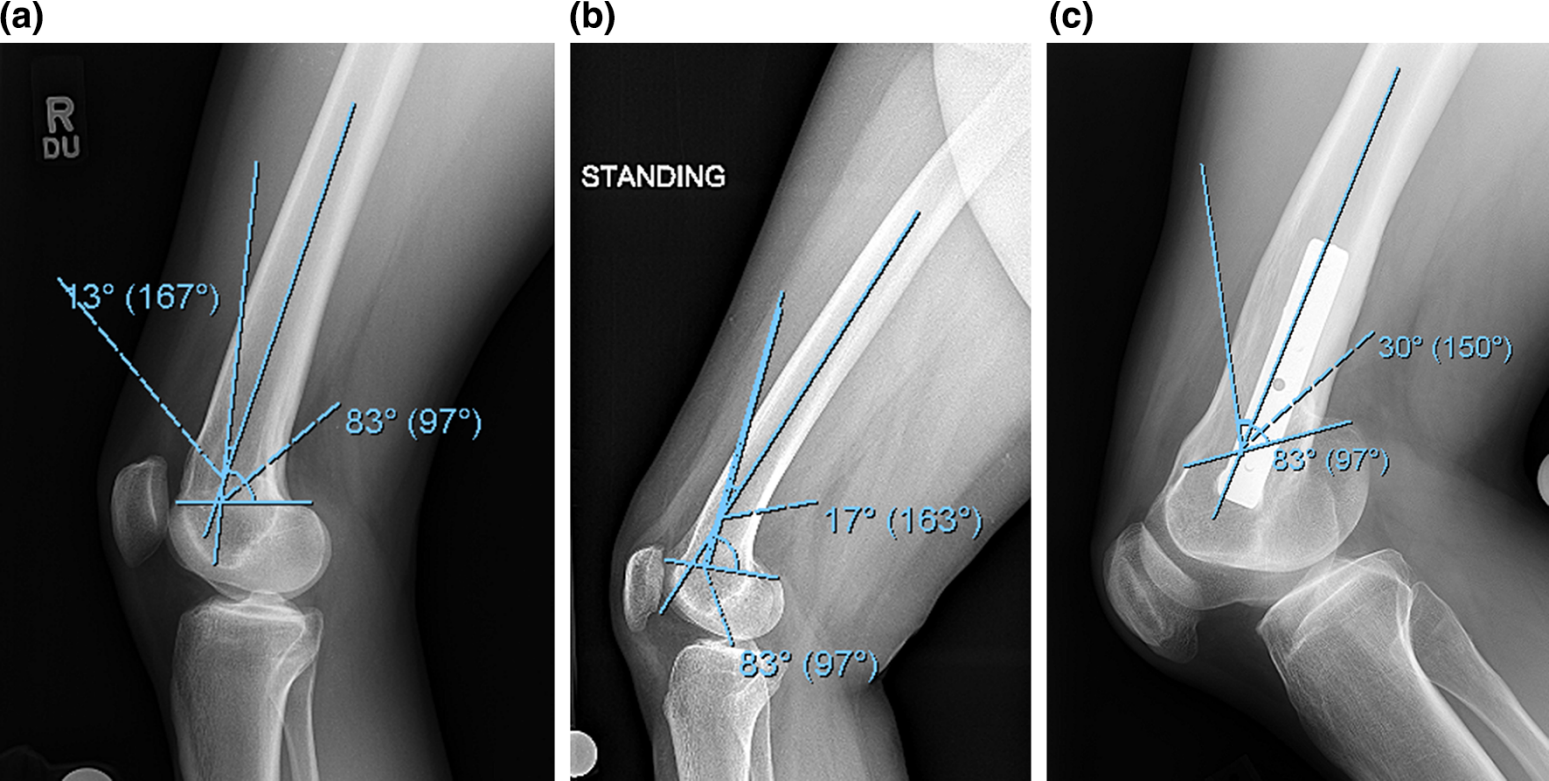
**a** The lateral radiograph of patient 1 shows the apex of deformity lies just proximal to the posterior condyles and has a magnitude of 13°, **b** the lateral radiograph of patient 2 shows the apex of deformity lies slightly more proximal than patient 1 and has a magnitude of 17°, **c** the lateral radiograph of patient 3 shows the apex of deformity lies at the level of the posterior condyles and has a magnitude of 30°

Three patients presented between 2014 and 2016 with complaints of a knee flexion deformity, shortening of the femur, with hip, knee, and low back pain. All patients had sustained an injury to the right femur during adolescence that involved damage to the distal femoral physis (Table 1). The resultant growth disturbance created the identical deformity pattern of distal femoral procurvatum and shortening (Fig. 1a, b). Patients underwent physical examination, including prone rotational profile and radiographic evaluation. The average knee extension loss was 12°, leg length discrepancy (LLD) was 47 mm, posterior distal femoral angle (PDFA) was 65°, and mechanical axis deviation (MAD) was 2 mm lateral (Table 2). Deformity planning indicated that the apex of the deformity was at the level of the distal epiphyseal scar in all cases (Fig. 2a–c). The magnitude of bony deformity was considered in the context of the physical exam. In case 3, for example, the patient lacked 15° of knee extension but had a bony deformity of 30°. The bone was corrected by 15° to achieve full knee extension without hyperextension.

**Table 1:** Patient injury details

Patient	Age at the time of injury (years)	Mechanism of injury	Nature of fracture	Initial treatment
1	13	Soccer	Distal femur growth plate closed SH	Closed reduction and casting
2	11	Motor vehicle accident	Distal femur growth plate closed SH	Closed reduction and casting
3	9	Motor vehicle accident	Distal femur growth plate closed SH	Closed reduction, percutaneous pinning, and casting. Later, osteotomy and plating for deformity correction

*SH* Salter Harris (SH grade is unknown by patient history)

**Table 2:** Patient demographics

Patient	Age at surgery	Laterality	Sex	Max knee ext (deg)	LLD (mm)	PDFA (deg)	MAD (mm)	LDFA (deg)
1	25	R	Female	− 10	25	73	0	84
2	16	R	Female	− 12	40	70	3 lateral	94
3	35	R	Male	− 15	75	52	3 lateral	93
Average	25	R		− 12	47	65	2 lateral	90

Knee extension measurements were obtained through physical exam

*R* right, *Ext* extension (a “−” sign indicated a lack of knee extension = flexion deformity), *LLD* leg length discrepancy, *PDFA* posterior distal femoral angle, *MAD* mechanical axis deviation, *LDFA* lateral distal femoral angle (ref-Paley)

**Fig. 3:**
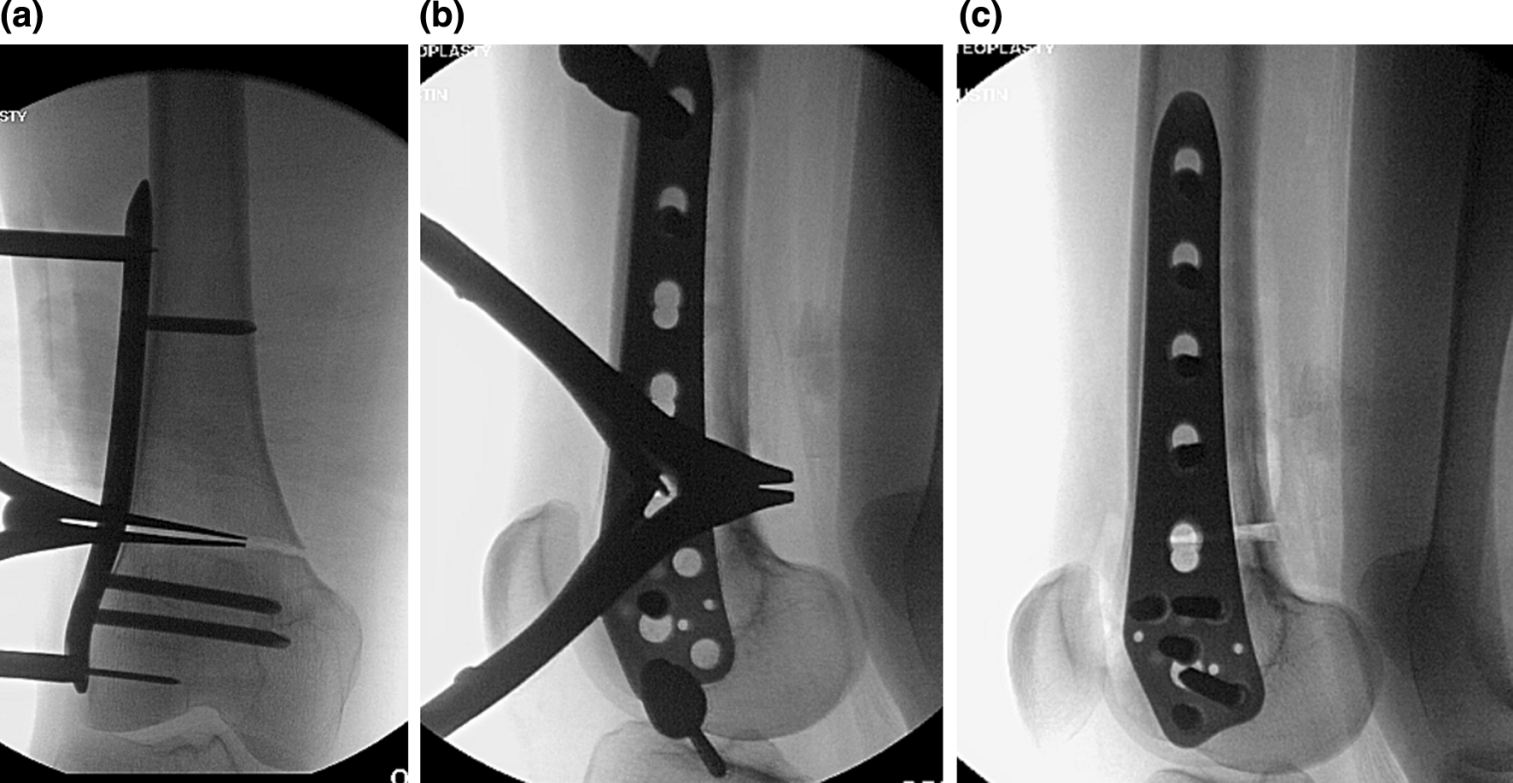
**a** An AP view of the posterior opening wedge osteotomy in patient 1 with the laminar spreader inserted centrally in the posterior cortex. Care is taken to avoid eccentric placement of the laminar spreader which could lead to unwanted varus–valgus deformity, **b** a lateral intraoperative view of the laminar spreader is seen holding the bony correction during plating, and **c** the final result of the posterior opening wedge correction after plating is seen on this lateral radiograph

**Fig. 4:**
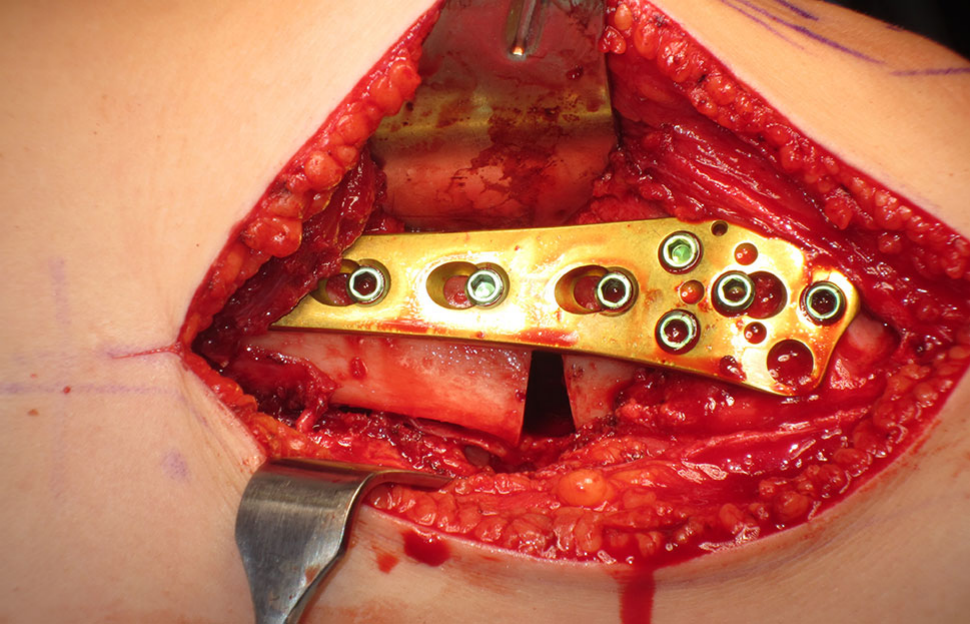
This intra-op radiograph from patient 2 shows the open posterior cortex prior to grafting with the plate secured

**Fig. 5:**
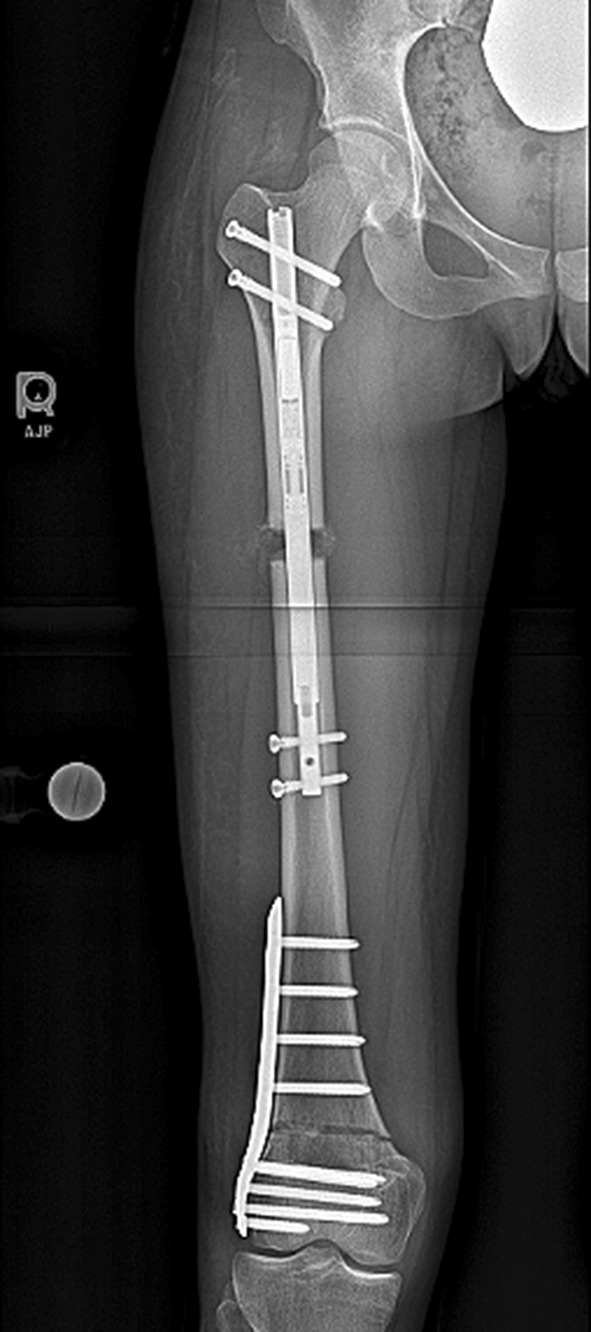
This post-operative radiograph demonstrates the entire construct including the piriformis entry ILN and the distal plate. This needs to be planned well to ensure the proper nail length and osteotomy location are selected so that the nail is long enough to control the lengthening bone but short enough to not interfere with the plate. The concern about a stress riser between the nail and the plate has not been an issue in this young adult population

**Fig. 6:**
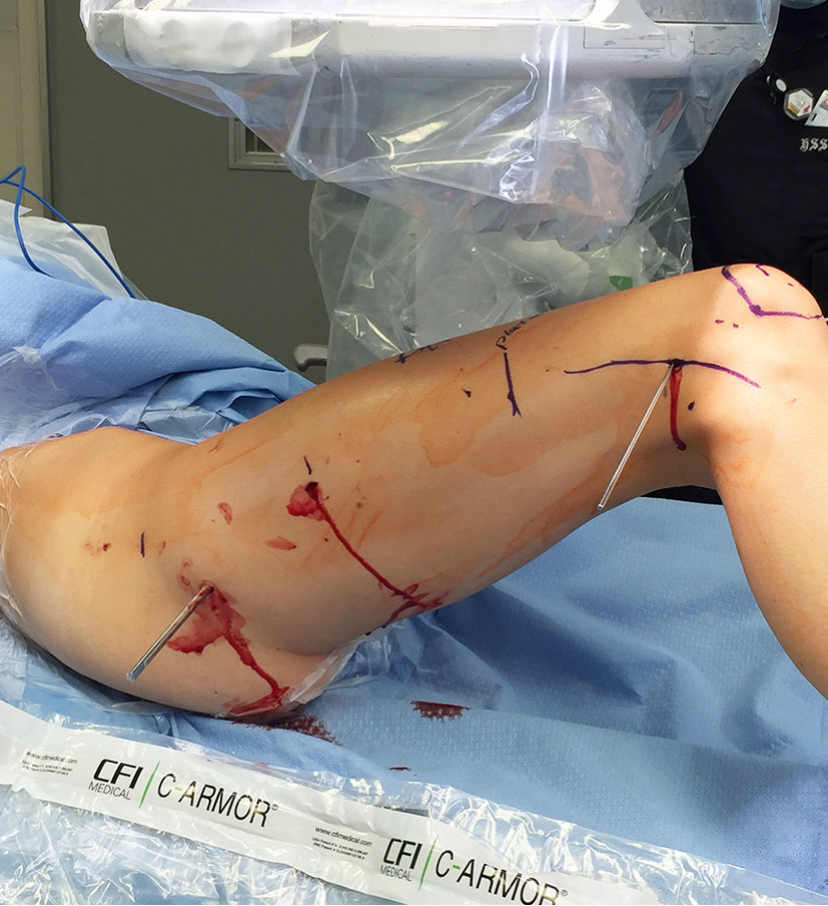
This intraoperative photograph shows the concept of using pins to mark the femoral rotation and, specifically, using a 6 mm half pin in the lesser trochanter to control the proximal fragment during nail preparation and insertion

One gramme of Tranexamic acid was administered intravenously at the time of the incision and 3 h later to prevent blood loss and haematoma. The deformities were corrected with an acute, posterior opening wedge osteotomy of the distal femur at the level of the trochlea and posterior condyles (a more distal osteotomy was not possible without cutting into the trochlea or condyles). The anterior cortex was maintained intact to increase the stability of the osteotomy site. Maintaining an intact anterior cortex was thought to prevent the introduction of coronal plane deformity and improve healing of the osteotomy. The posterior soft tissue was carefully dissected with the knee flexed. The osteotomy was performed with a micro-saw and osteotomes, ensuring division of the medial cortex. A laminar spreader was used to open the posterior cortex and correct the deformity (Fig. 3a–c). An anteriorly based closing wedge osteotomy could have been used as well but we were more comfortable with the opening wedge method. The osteotomy was then stabilized with a TomoFix (Synthes, West Chester, PA) titanium lateral plate and screws and grafted with allograft cancellous freeze-dried chips and demineralized bone matrix putty (Fig. 4). The femoral rotation was then marked with half pins. Two 6-mm pins were placed separately in the lesser trochanter and into the distal femur, posterior to the plate. These pins were used to control the fragments after osteotomy and set the rotational alignment. A second osteotomy was made proximally in the femur using a percutaneous technique employing multiple drill holes followed by a corticotomy. The Precice (NuVasive, San Diego, CA) internal lengthening nail was inserted using a piriformis fossa entry portal (Fig. 5). The ILN was 10.7 or 12.5 mm diameter and 245–275 mm in length. The length of the nail was selected pre-operatively and based on the position of the plate. The longest nail that would not touch the screws within the plate was utilized. The site of the osteotomy needed to be 8 cm+ the proposed lengthening (in centimetres) proximal to the distal tip of the nail. This would ensure that enough of the thick portion of the nail remained in the distal femur at completion of lengthening. In Case 2, an acute rotational correction of 15° was performed, correcting a retroversion malunion (Fig. 6). This was assisted with the posterior half pins inserted previously.

Post-operatively Rivaroxaban was started on day 2 and continued for 2 weeks to prevent VTE as per our post-limb deformity surgery protocol. Instructions for magnet use were taught to the patients during the admission. Lengthening began on post-operative day 4. It was done at 0.33 mm four times per day for 4 days and then three times per day until the desired length was obtained.

Patients were allowed 30 lbs. weight bearing immediately after surgery as determined by the plate rather than the ILN which allows 50–70 lbs. Weight-bearing restrictions were maintained for 3 months at which point the distal femoral osteotomy was healed; thereafter, the quality of proximal regenerate became the determining factor. Once two cortices of bone were seen on orthogonal X-rays, then weight bearing as tolerated was allowed.

**Fig. 7:**
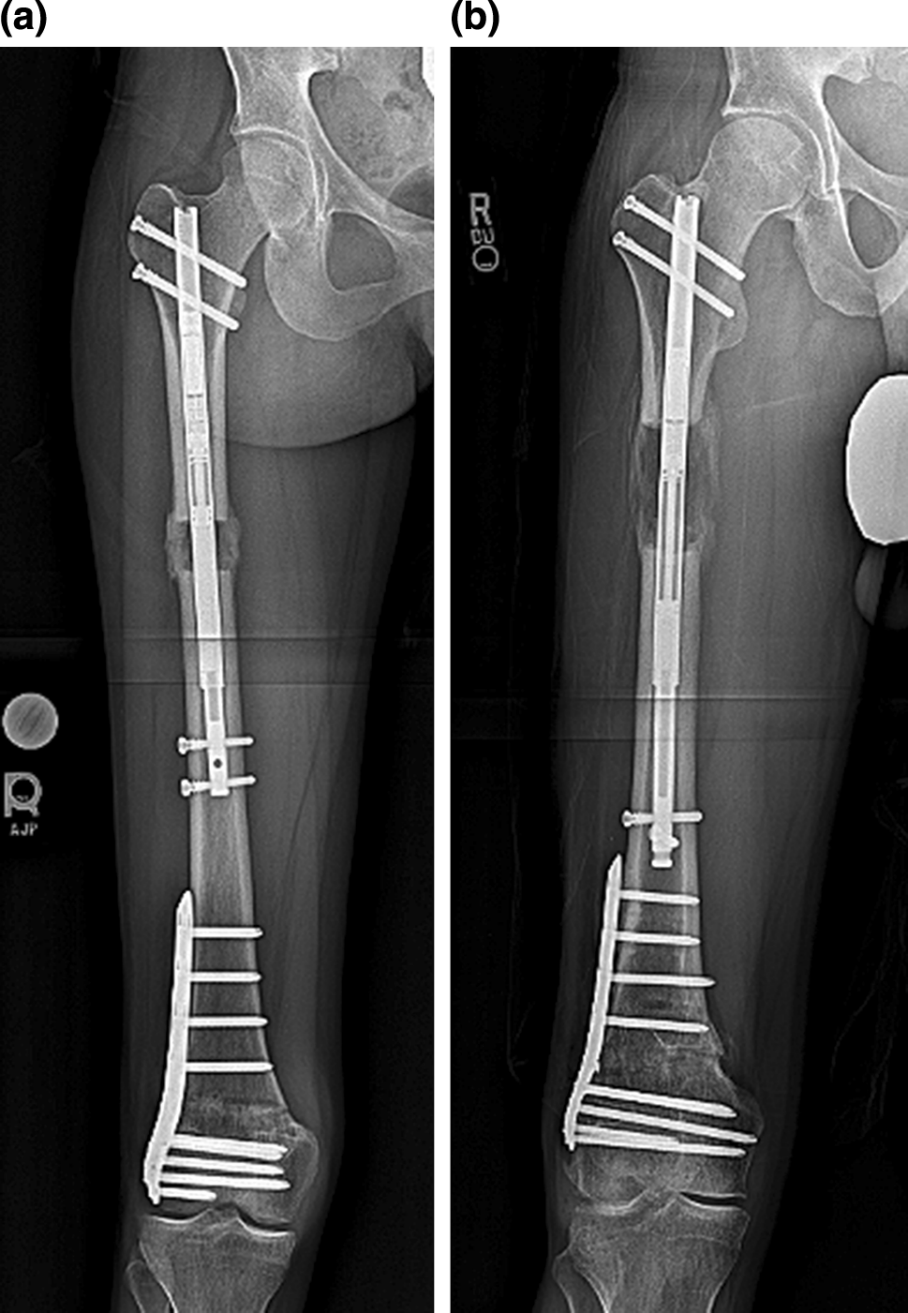
**a** This post-operative radiograph of patient 1 shows a consolidating proximal regenerate with bicortical bridging callus during the consolidation phase of lengthening, **b** this post-operative X ray of patient 3 shows a healthy regenerate during the distraction phase of lengthening. The rate of distraction was not altered after this appointment

Follow-up visits were conducted every 2 weeks with radiographs and the quality of the regenerate assessed (Fig. 7a, b). In Case 3, it was noted that the amount of bone lengthening was less than expected on post-op day 30 and the patient at risk for premature consolidation. The patient admitted to missing several adjustments and to smoking marijuana daily. He then proceeded with 0.99 mm per day lengthening with normal regenerate formation.

The primary outcome measures were: (a) an ability to achieve full knee extension; (b) the PDFA, MAD and the LLD measurements and; (c) the BHI (bone healing index). The MAD and PDFA measurements were made on 51 in., standing, bipedal radiographs taken 10 feet away from the patient and using a calibration ball. Outcome scores used the Knee Injury and Osteoarthritis Outcome Score (KOOS) [22]. Although the KOOS score is used primarily for knee osteoarthritis, it was selected in this study since it measures knee disability from acute injury as well. For a patient without arthritis, it is possible to score 100% once the acute injury has resolved. Complications including infection, a need for blood transfusion, knee stiffness, nonunion, compartment syndrome, and malunion were recorded.

## Results

Outcomes were divided into functional, implant-related, and bone (radiographic) results. Follow-up was 19 months (range 12–33). The average knee extension gain was 12° with all patients achieving full extension and none losing flexion (Table 3). Functional results, as measured by the KOOS, were excellent with average improvement in knee symptoms of 41%, knee pain of 29%, sports function of 32%, and quality of life 67% (Table 4). There were no implant-related failures.

**Table 3:** Knee ROM

Patient	Pre-knee ext (deg)	Post-knee ext (deg)	Pre-knee flex (deg)	Post-knee flex (deg)
1	− 10	0	140	140
2	− 12	0	130	130
3	− 15	0	120	130
Average	− 12	0	130	133

*ROM* range of motion, *Pre* pre-operative, *Post* post-operative, *ext* extension, *flex* flexion, *deg* degrees

**Table 4:** KOOS scores

Patient	Subscale items	Pre (%)	Post (%)
1	KOOS symptoms/stiffness	71.43	100
	KOOS pain	83.33	100
	KOOS function (daily living)	91.18	100
	KOOS function (sports and recreational actives)	55.00	100
	KOOS quality of life	37.50	100
2	KOOS symptoms/stiffness	85.71	100
	KOOS pain	80.56	100
	KOOS function (daily living)	82.35	100
	KOOS function (sports and recreational actives)	50	100
	KOOS quality of life	25	100
3	KOOS symptoms/stiffness	67.86	100
	KOOS pain	50	100
	KOOS function (daily living)	100	100
	KOOS function (sports and recreational actives)	100	100
	KOOS quality of life	37.5	100

Average	Subscale items	Pre (%)	Post (%)	Average improvement (%)
	KOOS symptoms/stiffness	58.92	100	41.08
	KOOS pain	71.29	100	28.71
	KOOS function (daily living)	91.18	100	8.82
	KOOS function (sports and recreational actives)	68.33	100	31.67
	KOOS quality of life	33.33	100	66.67

*KOOS* Knee Injury & Osteoarthritis Outcome Score

**Fig. 8:**
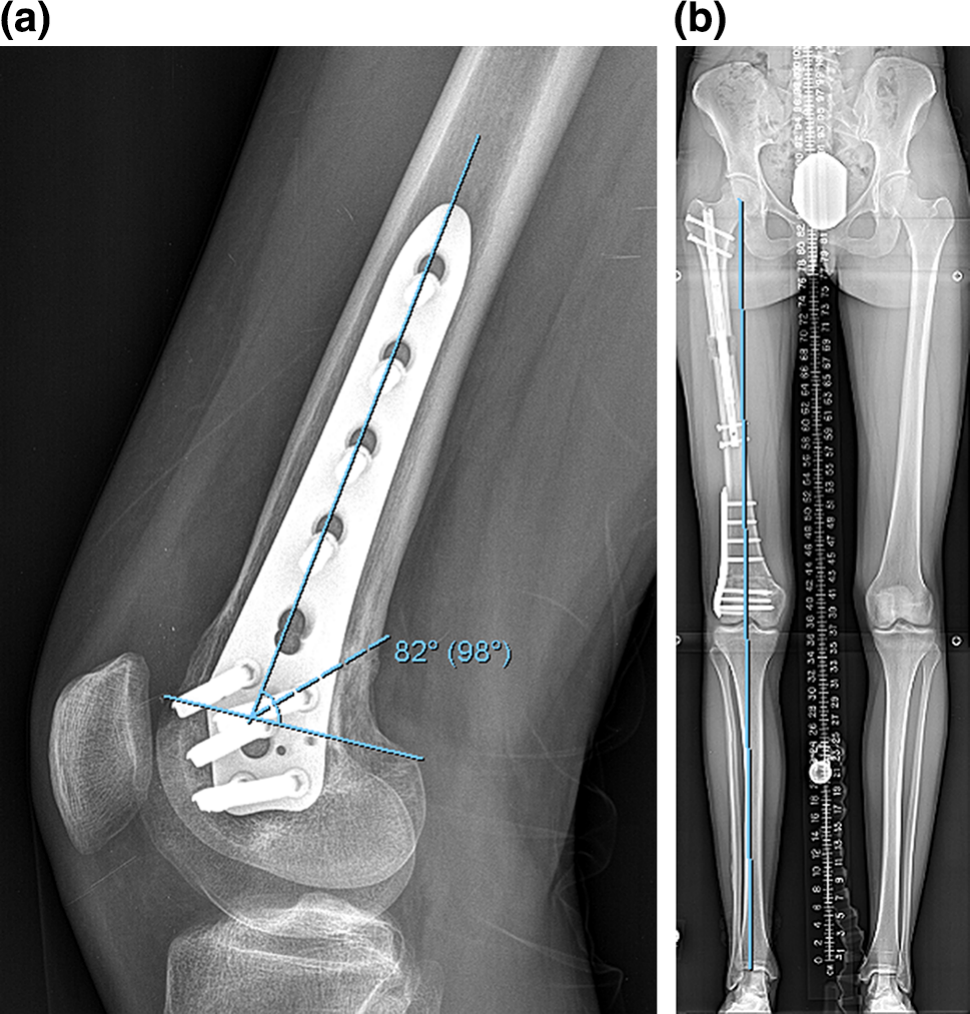
**a** This lateral femoral radiograph of patient 1 shows a PDFA of 82°, **b** this 51 in. standing film of patient 1 shows a MAD of 0 mm

Radiographic assessment showed the average amount of lengthening at the proximal osteotomy site was 40 mm (there was additional lengthening that occurred at the distal osteotomy site), and the residual LLD was 3 mm with the right leg shorter. The average post-op PDFA was 81° and MAD was 3 mm medial (Fig. 8a, b; Table 5). Bone healing index (days to consolidation from osteotomy date/cm lengthening) average was 24 days/cm (Table 5). All distal femoral osteotomy sites were healed at 3 months post-surgery. The distal osteotomy was located close to the knee joint. The average distance from the osteotomy to the knee joint line was 57 mm, and the distance from the osteotomy to the notch was 47 mm (Table 6).

**Table 5:** Radiographic results

Patient	Pre-LLD (mm)	Post-LLD (mm)	Proximal osteotomy length achieved (mm)	Distal osteotomy length achieved (mm) (calculated)	Pre-MAD (mm)	Post-MAD (mm)	BHI (days/cm)
1	25	2	21	2	0	0	26
2	40	3	34	3	3 lateral	0	28
3	75	5	65	5	3 lateral	9 medial	19
Average	47	3	40	4	2 lateral	3 medial	24

*Pre* pre-operative, *Post* post-operative, *LLD* limb length discrepancy, *MAD* mechanical axis deviation, *BHI* bone healing index

**Table 6:** Distance to distal femoral osteotomy from the knee joint line and from the supracondylar notch

	Dist (mm) to osteotomy from joint	Dist (mm) to osteotomy from notch
1	50	43
2	56	44
3	66	56
Average	57	47

Potential complications of post-operative anaemia with a need for blood transfusion, compartment syndrome, fracture of the femur between the plate and the tip of the IM nail, excessive stress on the distal osteotomy site from the proximal lengthening causing displacement of the distal osteotomy, venous thromboembolism (VTE), and knee stiffness were not seen.

## Discussion

The technique of two-level femur osteotomy stabilized with a DFO plate and ILN yielded a full range of knee motion which helped to provide improved outcome scores. Patients had excellent outcomes on the KOOS which improved an average of 8–66% depending on the subscale item (Table 4) All patients had knee pain and a lack of full extension pre-operatively which the KOOS was able to capture. They also complained of pre-surgery low back and hip pain during activities, presumably from the limb length inequality, which resolved completely after treatment.

**Fig. 9:**
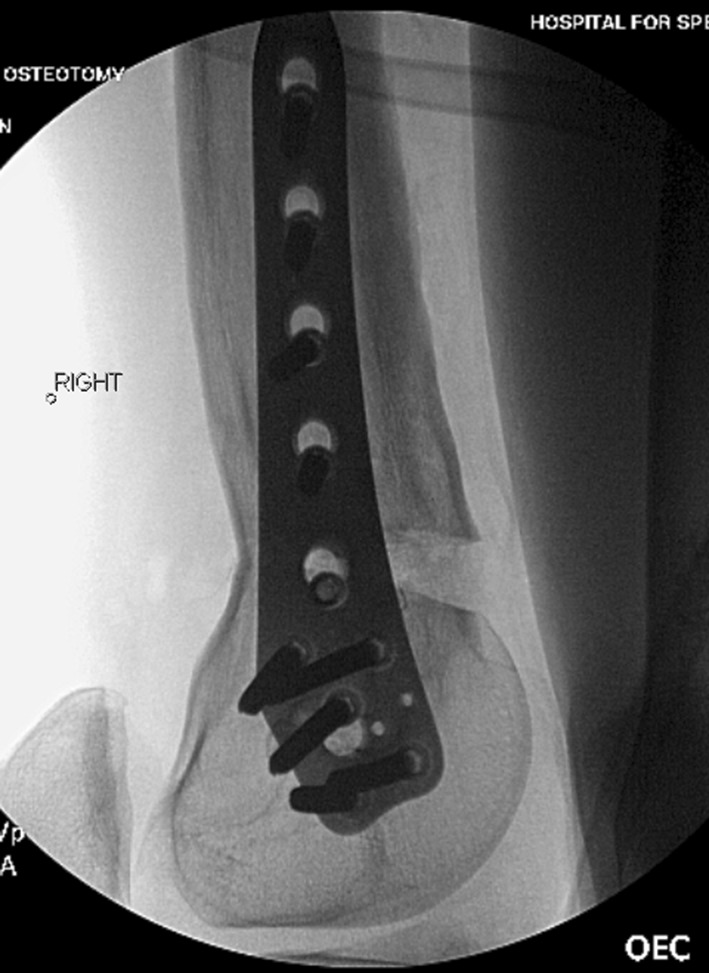
This intraoperative fluoroscopy film shows the obligate anterior translational deformity of the distal femoral fragment created by leaving the anterior cortex intact. This was felt to be clinically insignificant but was recognized

On radiographs the PDFA improved to the normal range and the average amount of clinical correction of knee flexion deformity was 12° with a maximum of 15° in patient 3. In that case the final lateral radiograph showed some anterior translational deformity of the distal fragment relative to the proximal fragment, which was without any negative functional impact, but suggests that a correction of a flexion deformity larger than 15° may be best accomplished by completion of the osteotomy through the anterior cortex, allowing for posterior translation of the distal fragment. A closing wedge technique may provide a better strategy as well (Fig. 9). The LLD decreased from 47 mm pre-op to 3 mm post-op as measured on standing radiographs. The patient with the largest residual LLD (5 mm, patient 3) was secondary to the patient feeling his limb was too long and refusing to do more lengthening. In all cases, the ILN was capable of more length and it was with the surgeon’s agreement to stop lengthening slightly short of the actual LLD to avoid the patient’s sense of over-lengthening. There was a minimal change in the MAD for patients 1 and 2. Patient 3 had a larger change in the MAD. This may have been due to an inability to accurately measure the actual distal femoral deformity (LDFA) pre-operatively secondary to the large flexion deformity and dysplastic distal femur. Alternatively, the large correction of flexion may have introduced varus into the distal osteotomy. The anterior cortex may have been too compromised (after 15° of opening) to maintain coronal plane alignment. The danger of a using a lateral approach, with lateral soft tissue stripping and insertion of instrumentation from the lateral side, was introducing an unintentional varus deformity.

The effect of lengthening the femur along the anatomic axis is to lateralize the mechanical axis [23], but this seemed to have no radiographic or clinical impact on outcome due to the modest amount of lengthening done in these cases. The BHI was low (24 days/cm), which was in keeping with observations from multiple studies on the ILN in femur reconstruction [21, 24, 25].

The average distance from the intercondylar notch to the distal osteotomy site was 47 mm. This is less than is considered the minimum acceptable distance for the ILN to succeed. Few studies on retrograde ILN have documented the distance of the distal femoral osteotomy to the intercondylar notch. One study reported an average distance of 81 mm [24]. Krieg et al. [25] explain that a limitation of retrograde ILN surgery is the need for a distance of 70–110 mm from the joint line to the osteotomy site, the variation being due to differences in femur length between patients. The authors add that an apex of deformity far distal from this osteotomy site may require excessive translation precluding use of the nail [25]. A review of our surgeries utilizing a single osteotomy with a retrograde ILN to correct distal femoral deformity yielded an average distance of 85 mm (with a minimum distance of 77 mm) from the osteotomy to the intercondylar notch. For the Precice ILN, the distance from the tip of the IM nail to the top of the most proximal distal locking hole is 48 mm. This would indicate that a minimum of at least 60 mm from the notch should be used to have both locking screws in bone with a margin of bone between the screw and the osteotomy to offer minimum control. It would be useful to define a zone of deformity in the distal femur (“a far-distal femoral deformity”) that lies between the joint line and, approximately, 70 mm proximally where an ILN will not have sufficient control of the distal fragment. This threshold needs to be further studied.

There were no true complications including need for additional surgery for knee contracture (a common problem after LON). The implant company guidelines require removal of the ILN which was done concomitantly with the plate removal. Patients did have pain over the lateral knee which was relieved by plate removal. The need to remove the plate due to pain could be considered a complication, but this was a possibility the patient was informed of prior to surgery.

## Conclusion

We recommend a two-level osteotomy for distal femoral deformities where the apex of deformity is very distal and is associated with shortening. An acute distal femoral osteotomy with plating will correct the sagittal plane deformity as was shown in this case series. A correction of over 12° may affect the coronal plane alignment and will cause some anterior translation of the distal fragment in the sagittal plane. Rotational correction can be performed acutely through the proximal osteotomy. It is unlikely the patient will need a blood transfusion if tranexamic acid is used intraoperatively and we no longer recommend cross-matched blood to be available. Knee range of motion will improve in extension and remain unchanged in flexion if the amount of lengthening is modest.
